# ﻿The complete chloroplast genome of *Rhododendronambiguum* and comparative genomics of related species

**DOI:** 10.3897/compcytogen.18.119929

**Published:** 2024-08-05

**Authors:** Wen Bao Ma, Yafei Ou, Buddhi Dayananda, Hui Juan Ji, Tao Yu

**Affiliations:** 1 Ecological Restoration and Conservation of Forests and Wetlands Key Laboratory of Sichuan Province, Sichuan, Academy of Forestry, Chengdu 610081, China Academy of Forestry Chengdu China; 2 School of Agriculture and Food Sciences, The University of Queensland, Brisbane, QLD 4072, Australia The University of Queensland Brisbane Australia; 3 Guizhou Provincial Key Laboratory for Rare Animal and Economic Insect of the Mountainous Region, Guiyang University, Guiyang 550005, China Guiyang University Guiyang China

**Keywords:** Chloroplast genome, molecular evolution, phylogenetic analysis, *Rhododendron*, *Rhododendronambiguum*

## Abstract

*Rhododendron* Linnaeus, 1753, the largest genus of woody plants in the Northern Hemisphere, includes some of the most significant species in horticulture. *Rhododendronambiguum* Hemsl, 1911, a member of subsection Triflora Sleumer 1947, exemplifies typical alpine *Rhododendron* species. The analysis of the complete chloroplast genome of *R.ambiguum* offers new insights into the evolution of *Rhododendron* species and enhances the resolution of phylogenetic relationships. This genome is composed of 207,478 base pairs, including a pair of inverted repeats (IRs) of 47,249 bp each, separated by a large single-copy (LSC) region of 110,367 bp and a small single-copy (SSC) region of 2,613 bp. It contains 110 genes: 77 protein-coding genes, 29 tRNAs, four unique rRNAs (4.5S, 5S, 16S, and 23S), with 16 genes duplicated in the IRs. Comparative analyses reveal substantial diversity in the *Rhododendron* chloroplast genome structures, identifying a fourth variant pattern. Specifically, four highly divergent regions (*trnI-rpoB*, *ndhE-psaC*, *rpl32-ndhF*, *rrn16S-trnI*) were noted in the intergenic spacers. Additionally, 76 simple sequence repeats were identified. Positive selection signals were detected in four genes (*cemA*, *rps4*, *rpl16*, and *rpl14*), evidenced by high Ka/Ks ratios. Phylogenetic reconstruction based on two datasets (shared protein-coding genes and complete chloroplast genomes) suggests that *R.ambiguum* is closely related to *R.concinnum* Hemsley, 1889. However, the phylogenetic positions of subsection Triflora Pojarkova, 1952 species remain unresolved, indicating that the use of complete chloroplast genomes for phylogenetic research in *Rhododendron* requires careful consideration. Overall, our findings provide valuable genetic information that will enhance understanding of the evolution, molecular biology, and genetic improvement of *Rhododendron* spieces.

## ﻿Introduction

The genus *Rhododendron* Linnaeus, 1753, comprising approximately 1000 species, stands as the largest genus within the family Ericaceae. It holds significant importance due to its high species diversity and broad distribution across the temperate regions of Europe, Asia, and North America (Chamberlain et al. 1996). These plants are renowned for their attractive flowers and foliage, and are extensively cultivated for their ornamental value, occupying a significant position in the realms of gardening and landscaping (Srivastava 2012). Moreover, *Rhododendron* species play a crucial role in montane ecosystems, hosting numerous dominant and ecologically significant species that contribute to the stability of plant communities in alpine or subalpine regions (Yu et al. 2017; Zhang et al. 2021). However, due to excessive deforestation and ongoing habitat deterioration, multiple *Rhododendron* species have been categorized as vulnerable on the IUCN Red List, including *R.protistumvar.Giganteum* Chamb. 1979, *R.redowskianum* Maximovich, 1870, and *R.aureum* Georgi, 1775 (Lu et al. 2021). Nonetheless, understanding of interspecific relationships and the timeline of diversification within the genus *Rhododendron* remains unresolved, largely due to the influence of natural hybridization and introgression (Ma et al. 2022b; Mo et al. 2022).

The molecular basis of *Rhododendron* species has been extensively explored in recent years, with nine *Rhododendron* genomes and various chloroplast genomes, including those of *R.calophytum* Franchet, 1886 and *R.delavayi* Franche, 1886, being published in recent studies (Li et al. 2020b; Shirasawa et al. 2021; Ma et al. 2022a; Shen et al. 2022; Li et al. 2023). The genetic information obtained has provided a range of genetic markers that have the potential to aid in reconstructing the phylogenetic connections among *Rhododendron* species. A comprehensive phylogenomic study of *Rhododendron* has been published recently, utilizing a partial chloroplast genome that encompasses 161 *Rhododendron* species (Mo et al. 2022). The advantages of utilizing the chloroplast genome include its moderate nucleotide substitution rates and the absence of homologous recombination, which makes it a valuable tool for elucidating relationships among *Rhododendron* species. This approach has provided a dependable taxonomic framework for the genus *Rhododendron*. However, only a limited number of studies have delved into the examination of chloroplast genome structure and variations within the genus *Rhododendron* (Li et al. 2020b; Shen et al. 2022). Since the comparison of *Rhododendron* chloroplast genomes not only establishes a crucial foundation for evolutionary investigations but also facilitates the exploration of fundamental chloroplast genome variability, there is a need for more comprehensive comparative studies utilizing the entirety of the chloroplast genome (Yu et al. 2021; Shen et al. 2022).

*R.ambiguum* Hemsley, 1911, belonging to subsection Triflora, is native to central and western Sichuan (China), thriving in thicket or woodland environments at elevations ranging from 2,300 to 3,300 meters, and occasionally reaching up to 4,500 meters (Shrestha et al. 2017). Functioning as an alpine species, *R.ambiguum* can be artificially cultivated and holds substantial horticultural significance. Studies have demonstrated the presence of abundant glycosides, terpenoids, and essential oils within *R.ambiguum*, indicating its promising potential for future commercial development (Shrestha et al. 2017). Currently, the genomic information available for *R.ambiguum* is rather limited. Furthermore, there is no complete nuclear genome data for subsection Triflora, and only a single chloroplast genome has been published (Shirasawa et al. 2021). The frequent occurrence of natural hybridization between subsection Triflora species and natural hybridization with other subgroups in the subgenus make it difficult to confirm and identify the phylogenetic location of species (Mo et al. 2022). The complete chloroplast genome will provide genetic data resources for the subsection Triflora and the whole genus *Rhododendron* phylogenetic analysis. In the current work, we report the characterization of the chloroplast genome of *R.ambiguum* and provide a comparative assessment alongside other relatives within the genus *Rhododendron*. This research focuses on (1) analyzing the chloroplast genome structural characteristics of *Rhododendron*, (2) identifying hypervariable regions and SSR loci that could serve as DNA barcodes in future, investigating the relative synonymous codon usage (RSCU), (3) gene Ka/Ks ratios in *Rhododendron* chloroplast genomes, (4) inferring the phylogenetic position of *R.ambiguum* within the genus *Rhododendron* using complete cp genome alignments.

## ﻿Material and methods

### ﻿Sample collection and DNA sequencing.

Fresh leaves of *R.ambiguum* were collected from Gongga Mountain National Nature Reserve in Sichuan, China (Longitude: 102.01791, Latitude: 29.77135, Altitude: 3291 meters). The leaves were immediately desiccated using silica gel for further analysis. The corresponding voucher specimens were deposited at the West China Subalpine Botanical Garden in Dujiangyan, Sichuan Province (Voucher No. 2019-GG-015). DNA was extracted from the leaves using a modified CTAB method (Doyle 1987). The purified genomic DNA was sequenced on an Illumina HiSeq X Ten platform by Biomarker Technologies, Beijing, China. Overall, we obtained a total of 8G paired-end reads after sequencing, according to previous studies, most *Rhododendron* genomes are about 650M, the sequencing depth in this research is about 18X (Shirasawa et al. 2021; Shen et al. 2022). The sequences were then trimmed using NGSQC (Dai et al. 2010) with default parameters.

### ﻿Assembly and annotation.

We assembled the sequences using MIRA software (http://sourceforge.net/apps/mediawiki/mira-assembler), with default settings, and NOVOplasty (Dierckxsens et al. 2016) using the reference genomes of *R.×pulchrum* (accession number: MN182619.1) and *R.calophytum* (accession number: OM373082.1) (Shen et al. 2020; Ma et al. 2022a). Genome annotation was performed using Cpgavas (Yong and Zheng 2012), complemented by manual curation and BLAST for gene verification. Circular chloroplast genome maps for *R.ambiguum* were generated using OGDRAW. The complete genome was uploaded to the National Center for Biotechnology Information with the GenBank accession number OR455462.

### ﻿Chloroplast genome boundaries comparison, divergence hotspot and SSRs analysis

The junctions between the chloroplast genomes of 18 Rhododendron species were examined using IRscope (Amiryousefi et al. 2018), which visualizes the expansion and contraction of inverted repeats (IRs) and the positioning of genes. Nucleotide diversity (Pi) was assessed through sliding window analysis using DnaSP v5 (Librado and Rozas 2009), with a step size of 200 bp and a window length of 600 bp. Simple sequence repeats (SSRs) were identified using MISA v1.0 with parameters set for various repeat units (Beier et al. 2017).

### ﻿Analysis of codon usage characteristics and selection pressure

Coding sequences from *Rhododendron* chloroplast genomes were extracted, and codon usage was analyzed using CodonW v1.4.2 (http://codonw.sourceforge.net). A clustered heatmap of Relative Synonymous Codon Usage (RSCU) values was generated at cloud.genepioneer.com. Selection pressures were calculated by determining nonsynonymous (Ka) and synonymous (Ks) substitution rates and their ratios using KaKs_Calculator v2.0 (Zhang et al. 2006), with non-applicable or infinite values defined as zero.

### ﻿Phylogenetic relationship reconstruction

The phylogenetic tree was constructed using Bayesian Inference (BI) methods, based on datasets of shared protein-coding genes (PCGs) and complete chloroplast genomes. Multiple sequence alignments were conducted using PhyloSuite (Zhang et al. 2020), with default settings. BI trees were inferred using MrBayes 3.2.6 (Huelsenbeck and Ronquist 2001) under a N/A model with 3,000,000 generations, discarding the initial 25% of sampled data as burn-in. The substitution models used were GTR+G+I for the PCGs dataset and GTR+G for the complete chloroplast genome dataset. *Vacciniumbracteatum* Thunberg, 1784, *V.macrocarpon* Aiton, 1789 and *V.uliginosum* Linnaeus, 1753 were selected as outgroups

### ﻿Availability of data and materials

The datasets generated or analyzed during the current study are available in the NCBI Bioproject repository OR455462.

## ﻿Results

### ﻿Characteristics of *R.ambiguum* chloroplast genome

**Figure 1.**
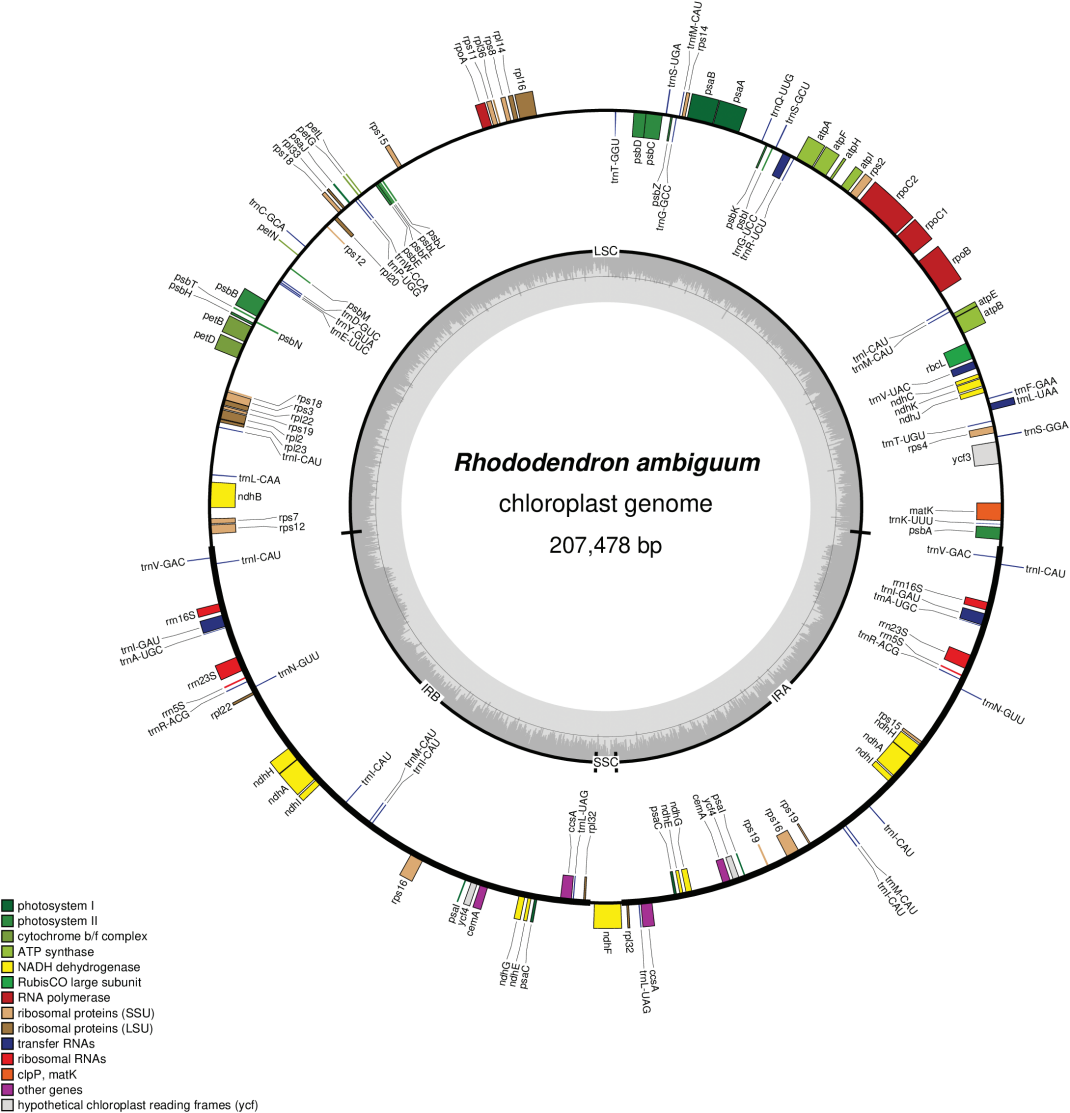
Chloroplast genome map of *Rhododendronambiguum*. Genes located outside of the circle were transcribed counter-clockwise, while genes shown inside were transcribed clockwise. The thick lines indicate the inverted repeat regions (IRa and IRb) that separate the genome into small (SSC) and large (LSC) single copy regions. Different genes were color coded.

The *R.ambiguum* chloroplast genome was typically circular quadripartite (Fig. 1). It spans a total length of 207,478 bp, comprising a pair of inverted repeats (IRs) of 47,249 bp each, separated by the large single-copy (LSC) region of 110,367 bp and the small single-copy (SSC) region of 2,613 bp. This genome encodes 110 unique genes, including 77 protein-coding genes, 29 tRNAs, and four unique rRNAs (4.5S, 5S, 16S, and 23S rRNA); 16 of these genes are duplicated in the IRs (Table 1). There are 14 genes with introns: ten protein-coding genes (*atpF*, *ndhA*, *ndhB*, *rpl2*, *rpl16*, *rps12*, *rps16*, *petB*, *petD*, and *ycf3*) and four tRNAs (*trnL-UAA*, *trnG-UCC*, *trnI-GAU*, and *trnV-UAC*). Notably, the *ycf3* gene contains two introns. The overall GC content of the *R.ambiguum* chloroplast DNA is 35.8%.

**Table 1.** Genes present in the *Rhododendronambiguum* chloroplast genome.

Group of gene	Genes name
Photostsyem I	*psaA*, *psaB*, *psaC*, *psaI*, *psaJ*, ycf3**, *ycf4*
Photostsyem II	*psbA*, *psbB*, *psbC*, *psbD*, *psbE*, *psbF*, *psbH*, *psbI*, *psbJ*, *psbK*, *psbL*, *psbM*, *psbN*, *psbT*, *psbZ*
Cytochrome b/f complex	petB*, petD*, *petG*, *petL*, *petN*
ATP synthase	*atpA*, *atpB*, *atpE*, atpF*, *atpH*, *atpI*
NADH dehydrogenase	ndhA*, ndhB*, *ndhC*, *ndhE*, *ndhF*, *ndhG*, *ndhH*, *ndhI*, *ndhJ*, *ndhK*
RubisCO large subunit	*rbcL*
RNA polymerase	*rpoA*, *rpoC1*, *rpoC*2
Ribosomal proteins (SSU)	*rps2*, *rps3*, *rps4*, *rps7*, *rps11*, rps12**^,T^, *rps14*, *rps15*, *rps16*, *rps18*, *rps19*
Ribosomal proteins (LSU)	rpl2*, rpl16*, *rpl20*, *rpl22*, *rpl23*, *rpl32*, *rpl33*
Other gene	*matK*, *ccsA*, *cemA*
Transfer RNAs	29 tRNAs (two contain a single intron)
Ribosomal RNAs	*rrn4.5*, *rrn5*, *rrn16*, *rrn23*

Note: a single asterisk (*) preceding gene names indicate intron-containing genes, and double asterisks (**) preceding gene names indicate two introns in the gene; ^T^ , trans-splicing of the related gene.

### ﻿Boundaries comparison and divergence hotspot analysis

**Figure 2.**
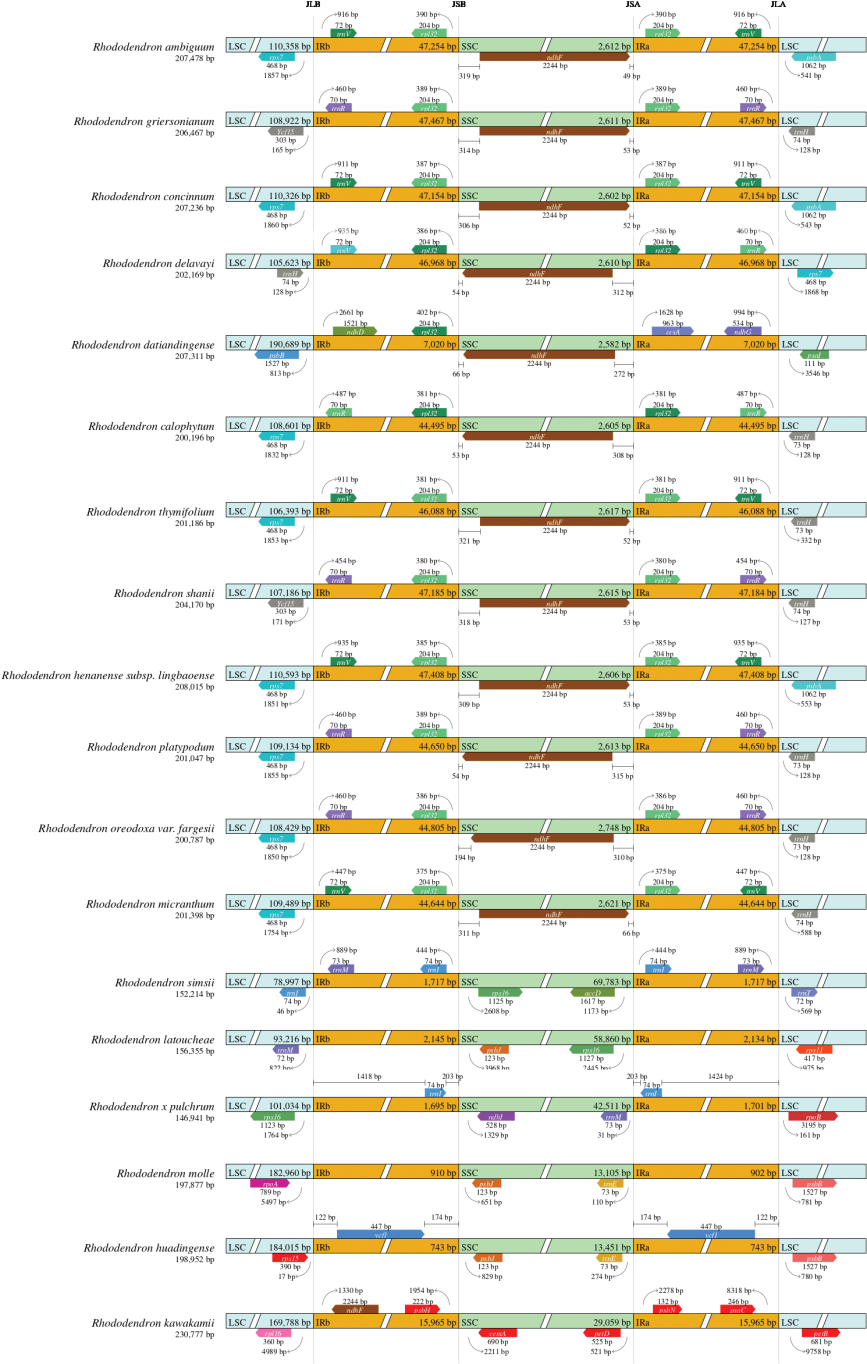
Comparison of the borders of the LSC, SSC and IR regions in 18 complete *Rhododendron* cp genomes. Genes transcribed forward were shown above the lines whereas genes transcribed reversely were shown below the lines. Gene lengths in the corresponding regions were displayed above the boxes of gene names.

We compared the complete chloroplast genomes of 18 *Rhododendron* species (Fig. 2), revealing a rich diversity in gene order within these genomes. Despite the overall retention of the quadripartite structure, the SSC regions exhibited large variations in length, displaying four main forms. The first category included 11 *Rhododendron* species with chloroplast genome lengths around 200,000 bp, where the SSC regions ranged from 2,582 bp to 2,748 bp, with the *ndhF* gene occupying more than 81% of this region. The second category consisted of three species (*R.simsii* Planchon, 1853, *R.latoucheae* Franchet, 1899, and *R.×pulchrum* Sweet, 1831) with shorter chloroplast genomes ranging from 146,941 bp to 156,355 bp. In *R.simsii*, the SSC region was notably large at 69,783 bp, only 9,214 bp shorter than the large single-copy (LSC) region. The third category included two species (*R.molle* Don, 1834 and *R.huadingense* Ding et Fang, 2005), which had the longest LSC regions and the shortest IRs, with the IRs comprising only 1% of the LSC region in *R.molle*. The fourth category, consisting of *R.kawakamii* Hayata, 1911 and *R.datiandingense* Tang et Zhuang, 2009, differed significantly from other species in terms of region length and gene arrangement.

**Figure 3.**
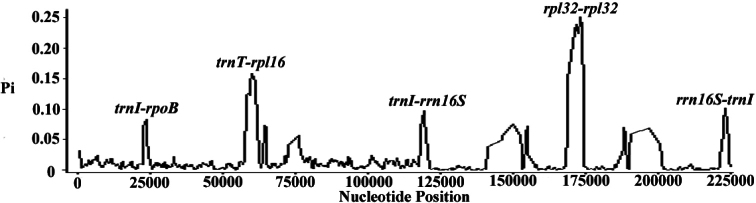
Sliding window analysis of *Rhododendron* chloroplast genome for nucleotide diversity (Pi) compared among 11 species similar structure, with window length 600 bp and step size 200 bp . Peak regions with a Pi value of > 0.08 were labeled with loci tags of intergenic spacers regions. X-axis: position of the midpoint of a window;Y-axis: nucleotide diversity of each window.

In terms of nucleotide variability (Pi), the first category exhibited ranges from 0 to 0.299, indicating high divergence (Fig. 3). We identified four highly variable regions (*trnI-rpoB*, *ndhE-psaC*, *rpl32-ndhF*, *rrn16S-trnI*) with Pi values ≥0.095. The *trnI-rpoB* and *ndhE-psaC* regions were located in the LSC region, *rpl32-ndhF* in the SSC region, and *trnI-rrn16S* in the IR region, highlighting that the LSC and SSC regions are more divergent than the IR regions. Overall, the non-coding regions were more divergent than the coding regions. These results underscore the potential of these regions as valuable phylogenetic markers for *Rhododendron* species.

### ﻿SSR analysis

**Figure 4.**
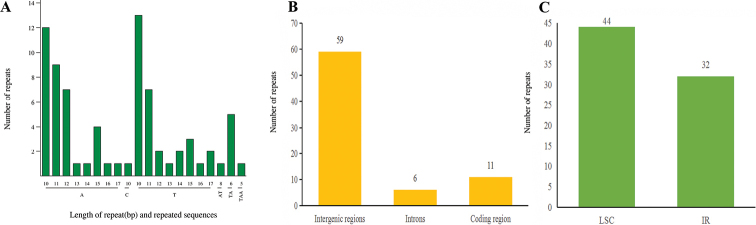
Distribution of SSRs in *Rhododendronambiguum* chloroplast genomes **A** length of repeat and repeated sequences **B** frequency of SSRs in the coding region, intergenic and intron region **C** frequency of SSRs in the LSC, IR and SSC regions.

In this study, a total of 76 SSRs with a repeat length of one to three bp were detected in *R.ambiguum* chloroplast genome. Among these SSRs, there were 69, six and one for mononucleotides (mono-), dinucleotides (di-), trinucleotides (tri-) nucleotide repeats, respectively, and tetranucleotides (tetra-), pentanucleotide (penta-) and hexanucleotide (hex) repeats were not found (Fig. 4A). The majority of the mononucleotides were composed of A/T, only one SSR was G/C, and total of the dinucleotides were AT/AT. Statistical analysis showed these SSRs were identified mainly in the intergenic regions (59), compared to six and 11 SSRs in the introns and coding region, respectively (Fig. 4B). In terms of distribution, 44 SSRs in LSC regions and 32 SSRs in IRs regions, no SSR was detected in the SSC region (Fig. 4C).

### ﻿Codon usage bias and gene selective pressure analysis

**Figure 5.**
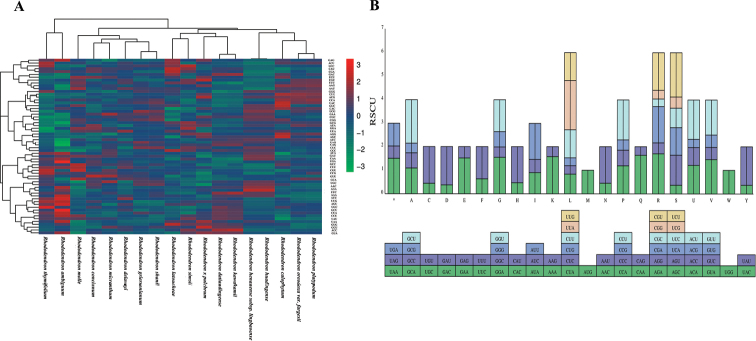
**A** Heat map analysis for codon distribution of all protein coding genes in 18 sequenced *Rhododendron* chloroplast genomes **B** codon content and codon usage of 20 amino acids and stop codons of all protein coding genes of *Rhododendronambiguum*.

The 18 *Rhododendron* chloroplast genomes were analyzed to investigate the relative synonymous codon usage (RSCU) (Fig. 5A). In this study, 64 codons are encoded 20 amino acids and one stop codon, The three most abundant amino acids were Leu (40,985), Ile (33,752) and Gly (27,610). The uncommon encoded amino acids were Ter (1,615), Cys (4,353) and His (8,417), respectively. Among these codons, RSCU value ranged from 0.317 (CGC) to 2.17 (UUA), and UUA had RSCU value between 2.06 to 2.17, indicating that Leu is preferentially coded by UUA in *Rhododendron* chloroplast genomes (Fig. 5B). Thirty codons showed average codon usage bias with RSCU > 1.00 in the chloroplast genes of *Rhododendron*, there were 13 codons ending in A (GCA,UCA,CCA,ACA,GUA,UAA,GAA,GGA,CGA,AAA,CAA.AGA and UUA), 16 codons ending in U (CUU, AGU, GGU, UUU, GUU, CAU, AUU, AAU, UGU, CGU, GAU, UAU, ACU, CCU, GCU and UCU), and 1 codon ending in G (UUG). This indicates that *R.ambiguum* prefers to end with A/U-ending codons.

**Figure 6.**
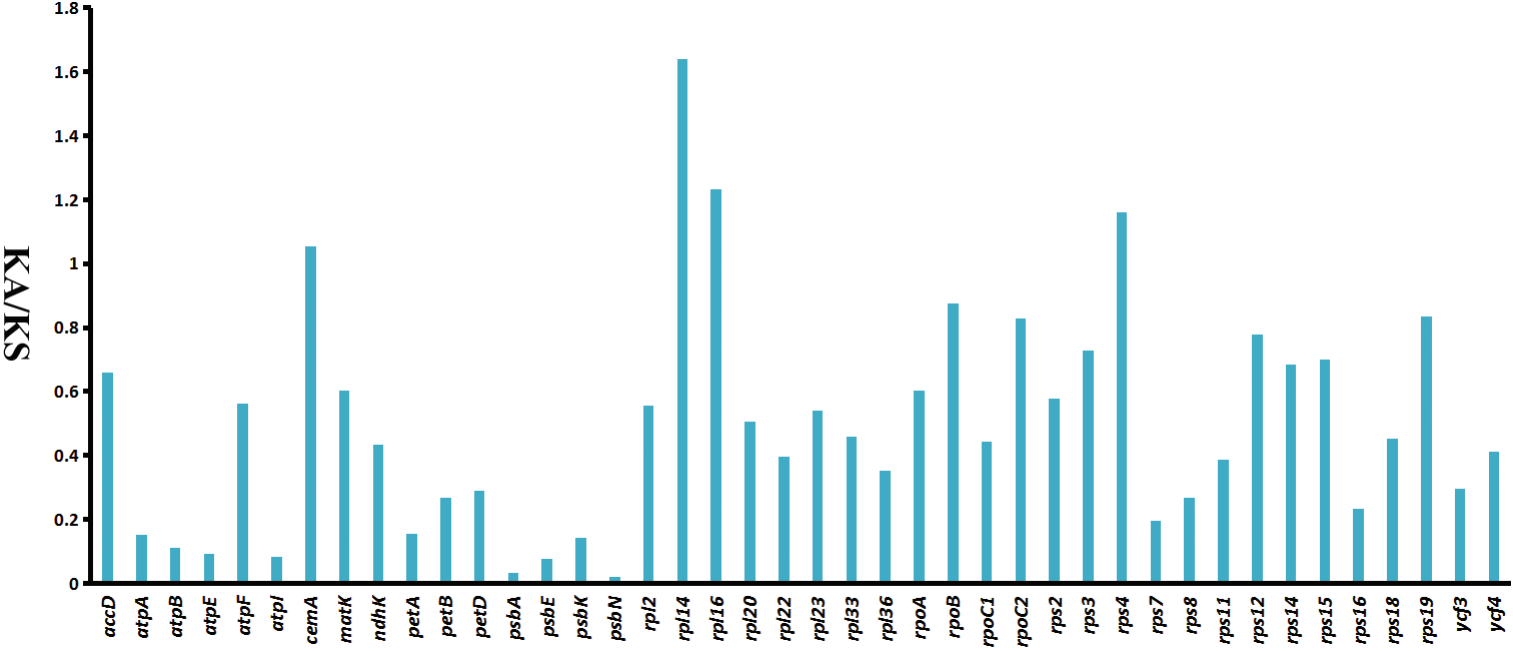
The Ka/Ks values of shared protein-coding genes of 18 *Rhododendron* chloroplast genomes.

The Ka/Ks ratio was calculated for 53 shared protein-coding genes between *Rhododendron* species cp genomes (Fig. 6). In all protein-coding genes of *Rhododendron*, *atpH*, *petG*, *psbD*, *psbF*, *psbL*, *psbJ*, *psbL*, *psbM*, *psbT*, *psbZ* and *rpl32* had no nonsynonymous rate, and four genes (*petG*, *psbL*, *psbT* and *rpl32*) had no synonymous substitution rates. The *rps15* gene had the highest synonymous rate (0.067) and highest nonsynonymous rate (0.073). The average Ka/Ks ratio analyzed in the 18 genomes was 0.496 for 42 protein-coding genes with Ka/Ks ratio, which were not region-specific. Most protein-coding genes showed purifying selection, 38 coding genes showed Ka/Ks ratios < 1, moreover, Ka/Ks ratios in the range of 0.5–1 has 15 protein-coding genes. The genes inferred to be undergoing positive selection were *cemA*, *rps4*, *rpl16* and *rpl14* (Ka/Ks ratios >1). The *rps4*, *rpl16* and *rpl14* were located in the LSC region, except the *cemA* in the IR region.

### ﻿Phylogenetic relationship reconstruction

**Figure 7.**
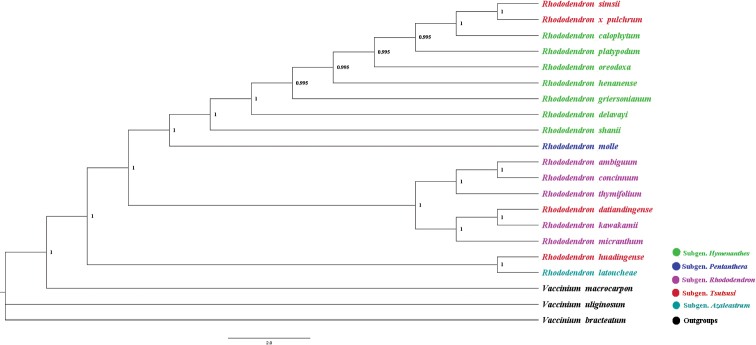
Phylogenetic relationships inferred by Bayesian Inference. This phylogenetic tree is based on shared protein-coding genes (PCGs) of 18 *Rhododendron* species and three outgroup species. Numbers at the nodes represent bootstrap support values.

Phylogenetic relationships among *Rhododendron* were estimated with two datasets using Bayesian inference (BI) methods. The BI tree inferred from shared PCGs confirms that *Rhododendron* species clustered three clades, and the boot-strap values of almost nodes were equal to 1 (Fig. 7). The shared genes and the whole chloroplast genome generated trees showed *R.ambiguum* close to *R.concinnum*, but the phylogenetic relationships of *Rhododendron* genus species were unresolved. The first clades showed that part of subgenus Tsutsusi, subgenus Hymenanthes Koch, 1872 and subgenus Pentanthera Don, 1834, species were clustered together. The second clades included species of subgenus Rhododendron and *R.datiandingense* Feng, 1996. belong to subgenus Tsutsusi in the middle position branch. The third clades composed of *R.latoucheae* Franchet, 1899 belong to subgenus Azaleastrum Maximovich, 1900 and *R.huadingense* Ding et Fang, 2005 belong to subgenus Tsutsusi. The results showed that confusion about phylogenetic position of subgenus Tsutsusi species was the main cause of the disorder phylogenetic tree. Different analyses based on the two datasets generated inconsistent topologies (Fig. 7, Suppl. material 1). In the datasets of chloroplast genome, different groups are not effectively divided indicating that PCGs were more efficient in determining phylogenetic relatedness rather than whole genomes, since the PCGs data exclude the influence of chloroplast genome structural variation.

## ﻿Discussion

The chloroplast genome of *R.ambiguum* exhibits a typical quadripartite structure, similar to most terrestrial plants, and spans 207,478 bp—slightly larger than the typical angiosperm chloroplast genome, which ranges from 120,000 to 160,000 bp (Fu et al. 2016). However, this size is average compared to the chloroplast genomes of closely related *Rhododendron* species. In this study, gene losses and large inversions were detected across *Rhododendron* chloroplast genomes, resulting in variations in genome length from 146,941 bp (*R.×pulchrum*) to 230,777 bp (*R.kawakamii*). Structural rearrangements within these genomes highlight extensive variations and significant expansions or contractions of inverted repeats (IRs) among *Rhododendron* species. Typically, the genes at the LSC/IRa/SSC/IRb boundaries are highly conserved within species of the same genus, with only slight expansions or contractions of IR regions observed (Turmel et al. 1999; Wang et al. 2021). Fig. 2 illustrates structural differences in the *Rhododendron* chloroplast genomes that exhibit a unique fourth variation pattern, underscoring the distinctive specificity of *Rhododendron* species, similar to findings in other studies (Li et al. 2020b; Shen et al. 2022). This unusual pattern in the *Rhododendron* chloroplast genomes may be influenced by factors such as genome mapping or annotation, in addition to natural factors.

Previous research has demonstrated that comparative chloroplast genomics is crucial for developing barcoding methods for species identification and advancing population genetics studies (Yuan et al. 2017; Li et al. 2020b). In our study, we identified variable regions within the chloroplast genomes of 11 *Rhododendron* species that exhibit similar structures. Notable differences were observed in regions such as *trnI-rpoB*, *ndhE-psaC*, *rpl32-ndhF*, and *rrn16S-trnI*. Notably, the *rpl32-ndhF* region has been previously utilized to test closely related species within the tribe Hydrangeeae (Granados Mendoza et al. 2013), and the *rrn16S-trnI* region has been extensively employed as a recombination site in chloroplast genome transformation research (Cui et al. 2014; Kaushal et al. 2020). Interestingly, the noncoding regions *ndhE-psaC* and *trnI-rpoB*, though never used in phylogenetic inference, have been recognized as hypervariable regions in monocots (de Santana Lopes et al. 2018).

Codon usage in the chloroplast genome protein-coding sequences of *Rhododendron* species is consistent, showing a preference for A/U-ending codons, similar to other plant genera (Silva et al. 2018; Shen et al. 2022). The amino acids Leu, Ile, and Gly are most frequently coded, which is a common pattern observed across various plant families. Notably, genes such as *cemA*, *rps4*, *rpl16*, and *rpl14* exhibit a relatively high Ka/Ks ratio (>1), suggesting positive selection. The *cemA* gene, encoding an envelope membrane protein, has shown signs of positive selection in the *Dalbergia* species (Wu et al. 2022). The ribosomal proteins *rps4*, *rpl16*, and *rpl14*, crucial for self-replication, have also been detected under positive selection in other taxa like Rosaceae and *Vicia* L., 1753 (Cheng et al. 2017; Li et al. 2020a). Given the diverse environmental conditions of *Rhododendron* habitats, from varying elevation gradients to light-related stress, this positive selection may indicate adaptive evolutionary responses.

Taxonomic determination within *Rhododendron* is challenging due to intermediate morphologies and hybrid origins between different species (Chamberlain et al. 1996; Ma et al. 2022a). Historically, phylogenetic analyses in *Rhododendron* have utilized markers such as ITS, retinol-binding proteins (RPB2I-1, RPB2I-2, RPB2I-3, RPB2I-4, RPB2I-5, RPB2I-6), and cpDNA loci (*atpB-rbcL*, *rbcL*, *matK*, *ndhF*, *psbA-trnH*, *trnL-trnF*, *trnL*, *trnT-trnL*, t*rnS-trnG*) (Kurashige et al. 2001; Shrestha et al. 2018; Mo et al. 2022), while elucidating some phylogenetic relationships, have also highlighted unresolved key nodes such as section Pentanthera to section Rhodora (60% bootstrap value) was indicated subgenus Mumeazalea Makino, 1914 and subgenus Azaleastrum Koch, 1872 section Choniastrum (59% bootstrap value)(Kurashige et al. 2001; Shrestha et al. 2018). The recent study by Mo et al. (2022) provides strong support for a stable and reliable taxonomic framework for *Rhododendron*, based on extensive chloroplast genome sampling. In Mo et al. (Mo et al. 2022) research revealed that *Rhododendron* species chloroplast genome failed to provide a complete circular structure, which the result file is several scaffold forms. As sequencing technologies advance, particularly with the introduction of third-generation sequencing, the accuracy of *Rhododendron* chloroplast genomes is expected to enhance their phylogenetic resolution significantly.

## ﻿Conclusions

This study assembled the complete chloroplast genome of *R.ambiguum*, revealing significant structural variations compared to other plant species. Key divergences were identified in four non-coding IGS regions, suggesting their potential as molecular markers for phylogenetic studies. The codon usage analysis showed a preference for A/U-endings, common across *Rhododendron* species. Notably, while most protein-coding genes exhibited purifying selection, genes such as *cemA*, *rps4*, *rpl16*, and *rpl14* showed signs of positive selection, indicating potential adaptation mechanisms to diverse environmental conditions. Phylogenetic analysis confirmed the close relationship between *R.ambiguum* and *R.concinnum* but also highlighted the complexity of relationships within the genus *Rhododendron*, underscoring the need for further research using complete chloroplast genomes. The insights gained from this study enhance our understanding of *Rhododendron*’s evolutionary biology and support the continued development of genomic resources for ecological and evolutionary studies.

## ﻿Author Contributions

HuiJuan Ji – methodology, Tao Yu – software, WenBao Ma and Yafei Ou – validation, WenBao Ma and Tao Yu – investigation, Tao Yu – writing and original draft preparation, Buddhi Dayananda – writing – review and editing, WenBao Ma – funding acquisition. All authors have read and agreed to the published version of the manuscript.

## ﻿Funding

This research was funded by InnovationTeam of Sichuan Province (no.LCTD2023CZ01-6), Natural Science Foundation of Sichuan Province (no. 2022NSFSC0136) and Natural Science Foundation of Sichuan Province (no. 2022NSFSC0094).

## ﻿Declarations

Dr. Wenbao Ma has obtained the permission by Sichuan Academy of Forestry to collect plant species from Gongga Mountain national nature reserve, Sichuan, China. Because of the important protective value of *Rhododendronambiguum*, Dr. Ma only collected a few leaves from the plants for further molecular study without damaging the plant growth. The plant material collection and experimental research were conducted according to the Plant Protection and Regulation of Sichuan Academy of Forestry.

The authors declare no conflict of interest.

## Supplementary materials

Supplementary material 1 Phylogenetic relationship was inferred used the Bayesian inference method based on whole chloroplast genome of 18 *Rhododendron* species and three outgroups Data type png https://binary.pensoft.net/file/1108385This dataset is made available under the Open Database License (http://opendatacommons.org/licenses/odbl/1.0/). The Open Database License (ODbL) is a license agreement intended to allow users to freely share, modify, and use this Dataset while maintaining this same freedom for others, provided that the original source and author(s) are credited.WenBao Ma, Yafei Ou, Buddhi Dayananda, HuiJuan Ji, Tao Yu

Supplementary material 2 Ka/Ks ratios > 1 genes among *Rhododendron* chloroplast genomes Data type xlsx https://binary.pensoft.net/file/1108386This dataset is made available under the Open Database License (http://opendatacommons.org/licenses/odbl/1.0/). The Open Database License (ODbL) is a license agreement intended to allow users to freely share, modify, and use this Dataset while maintaining this same freedom for others, provided that the original source and author(s) are credited.WenBao Ma, Yafei Ou, Buddhi Dayananda, HuiJuan Ji, Tao Yu

Supplementary material 3 Chloroplast genomes used for phylogenetic analyses Data type docx https://binary.pensoft.net/file/1108387This dataset is made available under the Open Database License (http://opendatacommons.org/licenses/odbl/1.0/). The Open Database License (ODbL) is a license agreement intended to allow users to freely share, modify, and use this Dataset while maintaining this same freedom for others, provided that the original source and author(s) are credited.WenBao Ma, Yafei Ou, Buddhi Dayananda, HuiJuan Ji, Tao Yu
