# Supplementary figures and images for: ﻿The complete chloroplast genome of Rhododendronambiguum and comparative genomics of related species
Source: Comp Cytogenet. 2024 Aug 5;18:143–59. doi: 10.3897/compcytogen.18.119929 (PMC11336383; doi:10.3897/compcytogen.18.119929)

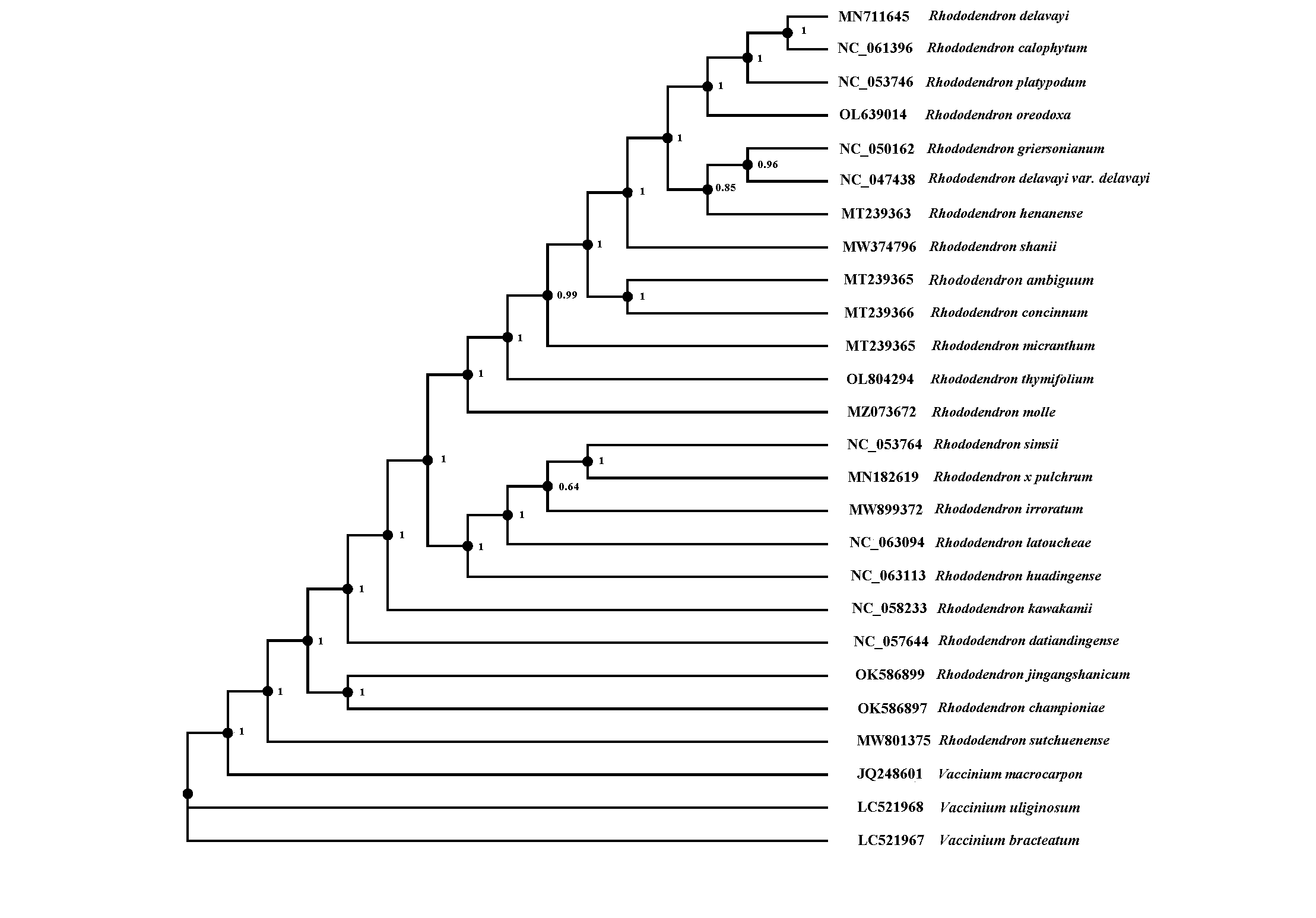

Supplement: Supplementary material 1 — Phylogenetic relationship was inferred used the Bayesian inference method based on whole chloroplast genome of 18 Rhododendron species and three outgroups [file comparative_cytogenetics-18-143_article-119929__-s001.png]
