# Supplementary material for: ﻿The complete chloroplast genome of Rhododendronambiguum and comparative genomics of related species
Source: Comp Cytogenet. 2024 Aug 5;18:143–59. doi: 10.3897/compcytogen.18.119929 (PMC11336383; doi:10.3897/compcytogen.18.119929)
Supplement: Supplementary material 3 — Chloroplast genomes used for phylogenetic analyses [file comparative_cytogenetics-18-143_article-119929__-s003.docx]

Table S2. Chloroplast genomes used for phylogenetic analyses.

| Species | GenBank accession no. |
| --- | --- |
| *Rhododendron calophytum* | NC_061396.1 |
| *Rhododendron concinnum* | MT239366.1 |
| *Rhododendron datiandingense* | NC_057644 |
| *Rhododendron delavayi* | MN711645 |
| *Rhododendron griersonianum* | NC_050162.1 |
| *Rhododendron huadingense* | NC_063113.1 |
| *Rhododendron henanense* | MT239363 |
| *Rhododendron kawakamii* | NC_058233.1 |
| *Rhododendron latoucheae* | NC_063094.1 |
| *Rhododendron micranthum* | NC_065477.1 |
